# Altered dopamine ontogeny in the developmentally vitamin D deficient rat and its relevance to schizophrenia

**DOI:** 10.3389/fncel.2013.00111

**Published:** 2013-07-17

**Authors:** James P. Kesby, Xiaoying Cui, Thomas H. J. Burne, Darryl W. Eyles

**Affiliations:** ^1^Department of Psychiatry, School of Medicine, University of California San DiegoLa Jolla, CA, USA; ^2^Queensland Brain Institute, University of QueenslandBrisbane, QLD, Australia; ^3^Queensland Centre for Mental Health Research, The Park Centre for Mental HealthWacol, QLD, Australia

**Keywords:** amphetamine, MK-801, dopamine, behavior, differentiation, development

## Abstract

Schizophrenia is a heterogeneous group of disorders with unknown etiology. Although abnormalities in multiple neurotransmitter systems have been linked to schizophrenia, alterations in dopamine (DA) neurotransmission remain central to the treatment of this disorder. Given that schizophrenia is considered a neurodevelopmental disorder we have hypothesized that abnormal DA signaling in the adult patient may result from altered DA signaling during fetal brain development. Environmental and genetic risk factors can be modeled in rodents to allow for the investigation of early neurodevelopmental pathogenesis that may lead to clues into the etiology of schizophrenia. To address this we created an animal model of one such risk factor, developmental vitamin D (DVD) deficiency. DVD-deficient adult rats display an altered behavioral profile in response to DA releasing and blocking agents that are reminiscent of that seen in schizophrenia patients. Furthermore, developmental studies revealed that DVD deficiency also altered cell proliferation, apoptosis, and neurotransmission across the embryonic brain. In particular, DVD deficiency reduces the expression of crucial dopaminergic specification factors and alters DA metabolism in the developing brain. We speculate such alterations in fetal brain development may change the trajectory of DA neuron ontogeny to induce the behavioral abnormalities observed in adult offspring. The widespread evidence that both dopaminergic and structural changes are present in people who develop schizophrenia prior to onset also suggest that early alterations in development are central to the disease. Taken together, early alterations in DA ontogeny may represent a core feature in the pathology of schizophrenia. Such a mechanism could bring together evidence from multiple risk factors and genetic vulnerabilities to form a convergent pathway in disease pathophysiology.

## INTRODUCTION

Schizophrenia is a severe and chronic psychiatric disorder consisting of a heterogeneous group of symptoms and cognitive impairments. On the basis of the convergent evidence from the fields of epidemiology, imaging and post-mortem analysis, the neurodevelopmental hypothesis and the dopamine (DA) hypothesis have become two major theories of schizophrenia. The developmental hypothesis proposes that genetic or environmental factors during critical early periods of brain development adversely impact on adult mental health (Murray and Lewis, 1987; Weinberger, 1987). The DA hypothesis proposes that DA dysfunction is central to the pathogenesis of schizophrenia (Carlsson and Lindqvist, 1963; Seeman and Lee, 1975; Angrist and Vankammen, 1984; Davis et al., 1991; Laruelle et al., 1996; Abi-Dargham et al., 1998). Recently, these two theories were revised and integrated through the substantial evidence that all the developmental risk factors which increase the risk of schizophrenia, appear to share a common endpoint or “final common pathway” of DA dysfunction (Di Forti et al., 2007; Murray et al., 2008; Howes and Kapur, 2009).

Clinical studies provide strong evidence of DA dysfunction in patients. Patients with schizophrenia show increased amphetamine-induced DA release in the striatum (Breier et al., 1997; Laruelle and Abi-Dargham, 1999; Laruelle et al., 1999; Abi-Dargham et al., 2009) and have altered presynaptic DA function, specifically increased DA synthesis capacity (Howes et al., 2012; Fusar-Poli and Meyer-Lindenberg, 2013). These factors are highly associated with psychosis (Howes et al., 2011a) and are generally not evident in stable schizophrenia patients that are not acutely experiencing a psychotic episode (Laruelle et al., 1999; Shotbolt et al., 2011). However, increased presynaptic DA function can also be observed during the prodromal phase of the disease (Howes et al., 2009) and in ultra-high risk subjects who then go on to develop psychosis (Howes et al., 2011b). Thus, it would appear that alterations in presynaptic DA function precede the onset of frank psychosis, suggesting that intervention prior to symptom onset may even offer the potential for preventing disease onset.

Substantial evidence from animal models indicates that fetal or perinatal factors can result in long-term alterations in dopaminergic function. For example, animal models designed to examine obstetric complications, such as fetal or neonatal hypoxia, resulted in increased DA-mediated behavioral responses, increased DA release and elevated basal DA in subcortical regions (Bjelke et al., 1991; Bernert et al., 2003; Boksa and El-Khodor, 2003; Decker et al., 2003). Rodent models with prenatal exposure to virus-like agents (e.g., the synthetic double-stranded RNA, Poly I:C) that explore the neurobiological correlates of maternal infection (Meyer et al., 2009) also exhibit increased levels of DA and DA metabolites and enhanced striatal DA turnover (Ozawa et al., 2006; Winter et al., 2009). In addition, heuristic evidence that early dopaminergic alterations can lead to impaired cognition and behavior later in life has also been described. For example, Kellendonk et al. (2006) have shown persistent deficits in cognition and altered DA function in a mouse model that transiently overexpresses DA 2 receptors in the striatum during development. Thus, abnormal DA signaling early in development would appear to produce long lasting impairments in brain function.

Evidence continues to mount from both epidemiological and pre-clinical studies to indicate that developmental vitamin D (DVD) deficiency may be also an important developmental risk factor for schizophrenia. Over the past decade, our studies on the DVD-deficient rat model firmly established that DVD deficiency affects brain cell proliferation, differentiation, and gross brain structure, it also produces long-lasting cellular changes and alterations in behavior in the adult offspring. In particular, the DVD-deficient adult offspring display enhanced DA-related behavioral responses and alterations in DA signaling. Understanding the mechanism of action linking DVD deficiency with altered DA signaling could provide clues to shared pathways underpinning the pathogenesis of schizophrenia.

In this article, we integrate findings derived from the DVD-deficient rat model and schizophrenia to propose that developmental DA dysfunction may be a core factor in the susceptibility and/or development of schizophrenia (**Box 1**). We begin with a concise summary of the epidemiological clues that suggest altered prenatal/perinatal vitamin D levels increase the risk of developing schizophrenia. Subsequently we introduce the DVD-deficient rat, discussing evidence from both early development and in adult offspring suggesting alterations in DA development and function. Furthermore, the multiple signaling pathways that may lead to such DA abnormalities in the DVD-deficient rat are also discussed. The final section reviews how DVD deficiency could lead to long-lasting neuroanatomical, neurochemical, and behavioral changes that are relevant to schizophrenia, and in particular, DA dysfunction.

**Box 1. Salient points.**

• *Schizophrenia*

Neurodevelopmental disorder associated with altered dopamine function both prior to and during disease onset

• *Developmental vitamin D (DVD) deficiency*

Associated with increased susceptibility to schizophrenia

• *DVD rat model*

DVD-deficiency in the rat leads to a pattern of altered developmental and adult dopaminergic function

• *Vitamin D and dopamine*

Early vitamin D signaling is intrinsically linked with the developing dopamine system

• *Animal models of schizophrenia*

Multiple animal models of schizophrenia show alterations in dopamine development prior to post-adolescent alterations in behavior

• *Dopamine and schizophrenia*

Although focus remains tied to dopamine as a common endpoint in schizophrenia, understanding common dopaminergic origins may be

## THE EPIDEMIOLOGY OF VITAMIN D AND SCHIZOPHRENIA

Numerous pieces of epidemiological evidence implicate low levels of maternal vitamin D as a potential risk factor for schizophrenia. Firstly, one of the most replicated findings is that people born in the winter and spring months of the year have an increased risk of developing schizophrenia later in life (Torrey et al., 1997b; McGrath, 1999; Davies et al., 2003), and this risk is larger at high latitudes that feature greater seasonal fluctuations (Davies et al., 2003). Secondly, people born in urban areas in comparison with those born in rural environments have an increased risk of developing schizophrenia (Torrey et al., 1997a; McGrath, 1999). Finally, the incidence of schizophrenia is significantly higher in the second generation of dark-skinned migrants to cold countries compared to native-born individuals (Cantor-Graae and Selten, 2005). Moreover, first generation migrants who arrive as babies or infants also have an increased risk of schizophrenia (Veling et al., 2011). This risk decreases with the increasing age of the migrant suggesting early life environmental conditions are critical. Given that vitamin D deficiency is common (1) during winter and spring, (2) at high latitudes (Holick et al., 1995), (3) in urban environments (McGrath et al., 2001) and (4) in dark skinned individuals (Clemens et al., 1982; Holick et al., 1995), these ecological data led to the hypothesis that low maternal vitamin D could be a modified risk factor for schizophrenia.

The direct analytical evidence in support of this hypothesis has also now been demonstrated. Schizophrenia has been shown to be ameliorated by vitamin D supplementation in the 1 year of life (McGrath et al., 2004). Most recently, and most importantly, a Danish population-based case-control study (423 cases and 423 control) that directly assessed vitamin D levels in blood spots from new born infants provided solid evidence showing that low prenatal vitamin D levels are associated with an increased risk of schizophrenia (McGrath et al., 2010). Taken together, these data support the hypothesis that an absence of vitamin D during development may lead to an increased risk of schizophrenia. To establish the biological plausibility of whether DVD deficiency could be related to schizophrenia, our group established a DVD-deficient rat model which has shown that low prenatal vitamin D adversely affects brain development and adult behavior, especially DA development and DA-related behaviors.

## THE DVD-DEFICIENT RAT MODEL OF SCHIZOPHRENIA

DVD-deficient offspring are produced by feeding female Sprague-Dawley rats a diet that lacks vitamin D but contains normal calcium and phosphorous. Rats are maintained on this diet for 6 weeks, after which and prior to mating, serum vitamin D_3_ depletion is confirmed by measuring the stable vitamin D metabolite 25 hydroxy-vitamin D (25(OH)D_3_) as <0.34 ng/ml (Eyles et al., 2009). Vitamin D deficient dams are maintained on the vitamin D depleted diet until the birth of pups. Control animals are kept under identical conditions but are supplied with standard rat chow containing vitamin D_3_. All dams (both control and depleted) are kept under standard housing conditions (control rat chow) after giving birth. Although vitamin D_3-_depleted dams and offspring remain normocalcemic, increased parathyroid hormone levels have been observed in both the dams and pups (Cui et al., 2010; Burne et al., 2011). In this model the exposure to vitamin D_3_ depletion is only transient as all dams are returned to a normal vitamin D containing diet ate birth. This is sufficient to replete DVD-deficient offspring to normal vitamin D_3_levels by two weeks of age. Importantly, calcium levels in vitamin D deficient dams and DVD-deficient pups are not altered by this protocol (Eyles et al., 2006; O’Loan et al., 2007). The acute effects of DVD deficiency including abnormal brain development, changes in gross brain structure and altered neurochemistry in addition to persistent alterations in behavior will be discussed in the following sections.

## BRAIN DEVELOPMENT IN THE DVD-DEFICIENT RAT

The idea that DA dysfunction represents the “final common pathway” in schizophrenia (Di Forti et al., 2007; Murray et al., 2008; Howes and Kapur, 2009) is supported by strong evidence of abnormal DA signaling in the *adult patient*, particularly at the presynaptic level (Howes et al., 2012; Fusar-Poli and Meyer-Lindenberg, 2013). However, we know little about the up-stream *developmental alterations* in DA physiology that may underpin these effects. For example, early life risk factors associated with schizophrenia may change the way DA systems develop. Thus, increases in presynaptic DA function in individuals who progress to clinical schizophrenia may result from abnormalities in the early ontogeny of DA systems. Animal models, such as the DVD-deficient rat, allow for more thorough investigations into early developing neurotransmitter systems and early developmental alterations that lead to behavioral and neurochemical abnormalities in the adult.

### EARLY DOPAMINERGIC ABNORMALITIES

Embryonic DA neuron development is a dynamic process with multiple factors responsible for normal function. In the rat, differentiation of monoamine cells in the substantia nigra (located in the midbrain) begins as early as embryonic day (E) 11 with the peak period of DA neuron birth occurring at E12(Lauder and Bloom, 1974; Gates et al., 2006). Subsequent innervation of the striatum in the basal ganglia from midbrain monoamine neurons occurs from E14-17(Voorn et al., 1988). DVD deficiency has been shown to alter the gene expression of key DA specification factors (i.e., factors involved in the phenotypic development of DA neurons) at both of these crucial time-points in DA development. At E12, coinciding with monoamine cell differentiation, expression of Nurr1 and p57Kip2 were decreased in DVD-deficient rats (Cui et al., 2010). Tyrosine hydroxylase (TH; the rate limiting enzyme in DA synthesis and a reliable marker of DA neurons) also appeared to be reduced in DVD-deficient embryos at this same time point. Nurr1 expression was also decreased at E15. This represents a period when dopaminergic innervation of the striatum begins to occur. Nurr1 (also known as NR4A2), an orphan nuclear receptor, is an essential factor in DA neuron development and maturation (Wallen et al., 2001) and p75Kip2 cooperates with Nurr1 during DA cell development (Joseph et al., 2003). Nurr1-deficient mice show complete DA neuron agenesis (Zetterstrom et al., 1997) and Nurr1 has been shown to directly activate the TH promoter gene in cell cultures (Sakurada et al., 1999; Iwawaki et al., 2000; Kim et al., 2003). Therefore, decreased Nurr1 expression coupled with a trend for reduced TH expression strongly suggests decreased or delayed DA cell differentiation in DVD-deficient rats.

These alterations in DA specification factors during development in the DVD-deficient rat compliment alterations in DA turnover identified at birth. Under normal conditions, the majority of DA metabolism is through intra-neural oxidative deamination via monoamine oxidase (MAO) to produce dihydroxyphenylacetic acid (DOPAC). This is followed by a subsequent extra-neural *O*-methylation via catechol-*O*-methyl transferase (COMT) to form homovanillic acid (HVA; Westerink, 1985). DVD-deficient pups show a 45% reduction in brain COMT levels at birth (Kesby et al., 2009). Moreover, this reduction in COMT is associated with an increased ratio of DOPAC to HVA, suggesting altered DA turnover. COMT remains an interesting target for schizophrenia research with less efficient isoforms increasing the risk of schizophrenia when coupled with adolescent marijuana use (Howes and Kapur, 2009). Thus, DVD deficiency directly impacts on factors that are essential in early DA neuron development and embryonic DA turnover.

### GROSS BRAIN ANATOMY, MITOSIS AND APOPTOSIS

The initial absence of vitamin D also affects other, more general, aspects of brain development that do not directly relate to DA neurons. For example, gross brain architecture is different in that DVD-deficient pups have cerebral hemispheres that are longer but not wider than control pups (Eyles et al., 2003). Furthermore, when corrections were made for the altered shape of these brains, the lateral ventricles were larger but the neocortex thinner than in control pups. Enlarged lateral ventricles have been observed in patients with schizophrenia and represent one of the more replicated neuroanatomical findings in schizophrenia (Chua et al., 2007; Nakamura et al., 2007; Pagsberg et al., 2007). These brain anatomical changes were also associated with altered rates of cellular proliferation.

Vitamin D is known to be involved in the modulation of cellular proliferation and apoptosis in many tissues (Banerjee and Chatterjee, 2003; Dusso et al., 2005). In the developing brain, levels of vitamin D receptor (VDR) expression coincide with increasing levels of apoptosis and decreasing levels of mitosis (Burket et al., 2003) suggesting similar actions to that seen in peripheral tissue. Conversely, the absence of vitamin D in the embryonic brain results in increased levels of mitosis and decreased levels of apoptosis (Eyles et al., 2003; Ko et al., 2004). DVD deficiency also altered gene expression profiles regulating mitosis and apoptosis in the brain (Ko et al., 2004). Furthermore, neurosphere cultures derived from DVD-deficient rat pups result in a greater number of neurospheres than cultures from control rat pups (Cui et al., 2007), also suggesting increased cellular proliferation. Thus at both the cellular and transcriptional levels, vitamin D appears fundamentally involved in the rate of proliferation and cell death in the brain. These early alterations in both DA signaling, brain structure and proliferation in DVD-deficient offspring appear to produce associated abnormal neurochemistry and behavior in adulthood.

## ALTERATIONS IN ADULT DVD-DEFICIENT OFFSPRING

### DOPAMINE-BASED ALTERATIONS

Subcortical DA function is an essential factor with regard to novelty-induced behavioral activation (Hooks and Kalivas, 1995) and both novelty and stress (i.e., handling etc.) result in increased DA release in the prefrontal cortex (PFC; Feenstra et al., 1995). Moreover, enhanced responsiveness to novelty is associated with an increased response to agents that enhance synaptic DA levels (Chefer et al., 2003). Adult DVD-deficient rats show enhanced novelty-induced locomotion on a range of tasks including the hole board and elevated plus maze (Burne et al., 2004; Kesby et al., 2006). Interestingly, this enhanced response can be attenuated in DVD-deficient rats with handling procedures and injections (Burne et al., 2006; Kesby et al., 2006).

Amphetamine has been shown to induce psychotic-like phenotypes in non-psychotic individuals and schizophrenia patients show enhanced DA release and positive symptoms relative to healthy individuals after exposure to low doses (Janowsky et al., 1973; Lieberman et al., 1987; Laruelle et al., 1999). Amphetamine-induced behaviors in rodents are therefore considered a model of the psychotic symptoms seen in schizophrenia. Amphetamine induces DA release in the brain primarily due to actions at the DA transporter (DAT; Sulzer et al., 1993; Wieczorek and Kruk, 1994; Jones et al., 1998). Female DVD-deficient rats show an increased sensitivity to amphetamine-induced locomotion as adults but not juveniles (Kesby et al., 2010). Although male DVD-deficient rats do not show an enhanced response after an acute dose of amphetamine, a similar sensitivity to amphetamine appears to occur after multiple doses (Kesby et al., 2010). Adult female DVD-deficient rats also have increased levels of DAT in the caudate putamen (CPu) and increased affinity for DAT ligands in the nucleus accumbens (Acb; Kesby et al., 2010) suggesting alterations in DAT function may mediate the enhanced response to amphetamine.

DVD-deficient rats also show increased sensitivity to the antipsychotic haloperidol (Kesby et al., 2006). Haloperidol is a typical antipsychotic used to treat the positive symptoms of schizophrenia and its antipsychotic potency is directly related to the blockade of DA 2 receptors (Seeman and Lee, 1975; Creese et al., 1976). DA 2 receptors however, do not appear to be altered in DVD-deficient rats (Kesby et al., 2010) indicating the behavioral response to haloperidol in DVD-deficient rats is more complex than a simple change in receptor density. Overexpression of DA 2 receptors in schizophrenia appears to have only a small effect size (Laruelle, 1998; Seeman and Kapur, 2000) and as such is not necessarily a key feature of the disease even though all antipsychotic drugs target these receptors. Thus, DVD deficiency induces persistent post-adolescent sensitivity to the behavioral effects of dopaminergic drugs that appears to mirror the post-adolescent onset of frank psychotic symptoms in schizophrenia patients and these sensitivities can be attenuated with the use of antipsychotic drugs.

Aspects of learning and memory are also affected in DVD-deficient rats. Latent inhibition refers to a learning phenomenon describing how it takes longer to associate relevance to a familiar stimulus than a novel stimulus. DVD-deficient rats have impaired latent inhibition (Becker et al., 2005) suggesting a deficit in the ability to attend selectively to relevant stimuli. Acutely psychotic patients also show impairments in latent inhibition (Gray et al., 1991; Lubow and Gewirtz, 1995) and DA agonists have been shown to decrease latent inhibition in healthy adult males (Swerdlow et al., 2003). Moreover, DVD-deficient rats show increased impulsivity and a lack of inhibitory control when assessed on the 5-choice continuous performance task (Turner et al., 2013). The increased impulsivity in DVD-deficient rats can also be attenuated with the atypical antipsychotic, clozapine. Impulsivity in healthy humans has been associated with the availability of the DAT (Costa et al., 2012) and in rats; DA receptors in the medial PFC also appear to be critical (Pardey et al., 2012). Thus multiple DA-based behavioral alterations are present in the adult DVD-deficient rat. However, other neurotransmitter systems, closely linked to the DA system, also appear to be affected in DVD-deficient rats.

### ALTERNATIVE NEUROTRANSMITTER SYSTEM ALTERATIONS

The use of N-methyl-D-aspartic acid (NMDA) receptor antagonists such as PCP, ketamine and MK-801 in animal models has become more widespread because the symptoms elicited in healthy people are more similar to those seen in people with schizophrenia than those observed after amphetamine (Krystal et al., 1994; Lahti et al., 2001). As a result, NMDA receptor hypofunction models of schizophrenia have been proposed and are also widely studied (Olney and Farber, 1995). DVD-deficient rats show a consistently enhanced locomotor response to MK-801(Kesby et al., 2006; O’Loan et al., 2007; Kesby et al., 2011). Importantly, this behavioral sensitivity is heavily dependent on the timing of vitamin D deficiency. Vitamin D deficiency in the later portion of gestation is required to elicit this behavioral sensitivity whereas vitamin D deficiency during the early portion of gestation has no impact (O’Loan et al., 2007). Coincidently, the later portion of gestation includes active DA neuron migration, differentiation and innervation in the embryonic brain. Although it is fairly clear that DA release is not required for the effects of MK-801 on locomotion (Carlsson and Carlsson, 1989), DA receptor antagonists have been shown to attenuate MK-801-induced behavior (Criswell et al., 1993; Willins et al., 1993; Andine et al., 1999; Kesby et al., 2006). Consistent with this, the enhanced locomotor response to MK-801 in DVD-deficient rats is selectively blocked by pretreatment with the DA 2 receptor antagonist haloperidol, at a dose that does not significantly attenuate MK-801-induced locomotion in control rats (Kesby et al., 2006). This suggests that abnormal DA signaling remains a component of the enhanced response to MK-801 consistently observed in DVD-deficient rats.

*In Summary*, DVD deficiency results in multiple outcomes in the adult animal that suggest neurotransmission and neuron integrity may be compromised. In addition, there is strong evidence that the altered response to psychomimetic drugs in adult DVD-deficient rats appears closely linked to DA function. The mechanism for how the developmental absence of vitamin D may influence DA signaling in the adult remains unknown. However given the multiple pieces of evidence indicating early alterations in the ontogeny of DA systems in this model we suspect this may hold the key.

## LINKING DOPAMINE ABNORMALITIES TO DVD DEFICIENCY

### EARLY VITAMIN D SIGNALING AND DOPAMINE

Vitamin D is a nuclear steroid. Its signaling is via a single nuclear receptor called the VDR which is expressed widely throughout the human (Sutherland et al., 1992; Zehnder et al., 2001; Eyles et al., 2005) and rat brain (Clemens et al., 1988; Fu et al., 1997; Prufer et al., 1999). The VDR shares structural characteristics with other nuclear steroid receptors (Mangelsdorf et al., 1995). After ligand binding the VDR forms a heterodimer with the retinoid X receptor (RXR). This complex binds to vitamin D response elements (VDRE) in the promoters of a number of genes; to regulate their transcription (Christakos et al., 2003). Expression of the VDR begins early in development (Fu et al., 1997; Veenstra et al., 1998; Erben et al., 2002; Burket et al., 2003; Cui et al., 2013) and increasing levels of VDR coincide with increasing levels of apoptosis and decreasing levels of mitosis (Fu et al., 1997; Veenstra et al., 1998; Erben et al., 2002; Burket et al., 2003). However, it is the coincident expression of the VDR within developing DA neurons (Cui et al., 2013) and projections in the brain that suggest an important role for vitamin D in the developing DA system.

#### Effects of Vitamin D on dopamine differentiation and innervation

Expression of the VDR can be found as early as E12 in the neuroepithelium (Veenstra et al., 1998) coinciding with the peak differentiation of monoamine cells in the substantia nigra; the primary source of midbrain dopaminergic projections to the basal ganglia (Lauder and Bloom, 1974; Gates et al., 2006). As dividing mesencephalic DA progenitor cells stop proliferating, they immediately begin to express specification factors [initially Nurr1 (Joseph et al., 2003) with p57Kip2 expressed soon after (Wallen et al., 1999)] that help to establish the neurotransmitter phenotype of these cells. DVD-deficient E12 embryos show decreased gene expression of Nurr1, p57Kip2 and TH (Cui et al., 2010) suggesting altered vitamin D signaling affects early monoamine cell development, perhaps even prior to E12. Not surprisingly, all three of these factors are linked and it would appear that Nurr1 is the upstream effector that results in altered p57Kip2 and TH levels. For example, Nurr1 has been shown to activate the expression of p57kip2 which then cooperates with Nurr1 in the maintenance of DA neurons (Joseph et al., 2003). Moreover, Nurr1 has been shown to regulate important proteins in DA synthesis and function including TH, vesicular monoamine transporter 2 (VMAT2) and DAT (Smidt and Burbach, 2007). Thus a decrease in Nurr1 expression would be expected to result in decreased p57Kip2 and TH as found in the DVD-deficient embryo.

Monoaminergic striatal innervation occurs from E14-17 (Voorn et al., 1988) with functional release observed at E18 (Nomura et al., 1981). Consistent with the premise that vitamin D plays a role in dopaminergic cell development, VDR expression in the differentiating field of the midbrain and basal ganglia can be observed by E15 (Veenstra et al., 1998). Furthermore, DVD-deficient embryos show decreased expression of Nurr1 at E15 (Cui et al., 2010). Thus, the appearance of the nuclear expression of the VDR in the mesencephalon at the peak period of DA neuron differentiation raises the possibility that the absence of vitamin D at this point may lead to changes consistent with the absence of this ligand. Namely, increased rates of DA neuron proliferation and delayed differentiation. This is consistent with the reduction in the expression of post-mitotic specification factors such as Nurr1. Interestingly, although Nurr1 gene expression in the mesencephalon peaks from E13 to E15 (Volpicelli et al., 2004) the levels of Nurr1 in the developing rat cortex show a different temporal window of expression with peak protein levels occurring later at P1 (Li et al., 2011). Whether the levels of cortical Nurr1 are decreased or delayed as observed in the mesencephalon of DVD-deficient rats is currently unknown.

How the absence (or presence) of vitamin D could alter Nurr1 levels remains unknown. However, retinoid function and specifically the interactions of retinoid receptors and Nurr1 have led researchers to suggest that retinoid signaling may be one link between the genetic and environmental susceptibility to schizophrenia (Palha and Goodman, 2006). Both Nurr1 and the VDR form heterodimers with the RXR (Mangelsdorf et al., 1995; Perlmann and Jansson, 1995; Aarnisalo et al., 2002). Indeed, signaling through the RXR-Nurr1 heterodimer is involved in the neuroprotective actions of Nurr1 in DA neurons (Wallen-Mackenzie et al., 2003). However, in rat neural precursor cells the RXR-Nurr1 heterodimer has been shown to *reduce* Nurr1 activity in DA neuron generation and *reduce* TH promoter activity (Yoon et al., 2010). It is important to note that levels of the VDR are unaltered in DVD-deficient pups (Eyles et al., 2003) allowing for ligand-independent actions. The interactions between, and functions of, the VDR and RXR appear to be extremely dependent on the presence of vitamin D. For example, the VDR-RXR heterodimer with no ligand acts as a weak transcriptional repressor (Tagami et al., 1998). Furthermore, when no ligand is present the RXR increases the nuclear accumulation of VDR by slowing nuclear export whereas, when bound to vitamin D, the VDR regulates the import of the RXR into the nucleus (Prufer and Barsony, 2002). Thus the non-ligand bound VDR may lead to decreased levels of cytosolic VDR and reduced competition for RXR compared with Nurr1. This may lead to an increase in the inhibitory functions of the RXR-Nurr1 heterodimer on DA neuron generation (Yoon et al., 2010). Decreased DA neuron generation would subsequently lead to reduced levels of Nurr1 and TH as found in DVD-deficient embryos. The presence and interaction of the VDR (minus ligand) on the availability or function of the RXR-Nurr1 heterodimer is unknown but this remains an intriguing target.

In addition, recent work also suggests that Nurr1 expression induces the expression of the glial derived neurotrophic factor (GDNF) receptor, Ret, in adult nigral DA neurons (Decressac et al., 2012). The decreased Nurr1 levels may therefore decrease GDNF signaling in DVD-deficient embryos. Moreover, evidence continues to accumulate indicating vitamin D positively regulates GDNF levels in the developing mesencephalon (Orme et al., 2013). GDNF is an important factor involved in DA neuron development, survival and function (Lin et al., 1993; Chun et al., 2002; Kholodilov et al., 2004). Thus, the combined local expression these three factors (VDR, RXR, and Nurr1) in addition to their cooperative signaling capabilities may be causal for the downstream effects of DVD-deficiency on DA neuron development.

#### Effects of Vitamin D on postnatal dopamine events

Developing DA neurons undergo two postnatal phases of natural cell death (Oo and Burke, 1997); the first peak is around postnatal day (P) 2 and the second peak occurs around P14. The combination of these two phases of cell death determines the number of DA cells within the adult brain. There are various regulatory factors involved in this process. For example, the first of these stages is heavily regulated by GDNF levels (Oo et al., 2003) and interactions with striatal targets (Burke, 2004). The second stage is less well understood but the dependence on striatal targets appears to remain (Burke, 2004). These processes establish DA neuron number and functional connectivity in the juvenile animal. Vitamin D has been shown to increase GDNF synthesis in the brain (Wang et al., 2000). Given the importance of GDNF in the postnatal programmed cell death of DA neurons in addition to its roles in DA neuron development, survival and function (Lin et al., 1993; Chun et al., 2002; Kholodilov et al., 2004); this represents a potential factor that could permanently alter key dopaminergic regions that contribute to adult behavior. We assume the levels of Nurr1 in DVD-deficient rats would have returned to control levels by this stage given the trend toward this in embryonic development (Cui et al., 2010). However, any consequence of earlier Nurr1 signaling deficits on the expression of the GDNF receptor, Ret, may persevere. Subsequently, this may lead to reductions in GDNF signaling during these crucial phases of DA cell death in DVD-deficient rats.

These DA cell death events are also dependent on the functional interaction of DA neurons and their striatal targets (Burke, 2004). Therefore, compromised neuronal signaling after DVD deficiency could potentially impact the survival of DA neurons. For example, silencing the VDR in cortical cultures from E16 embryos disrupts L-type voltage-sensitive calcium channels which are involved in calcium homeostasis and neuronal integrity (Gezen-Ak et al., 2011). Furthermore, DVD-deficient pups also show altered DA turnover as a result of decreased levels of COMT (Kesby et al., 2009). Both of these vitamin D-induced alterations could potentially lead to altered signaling in DA neurons. Thus altered DA signaling at birth suggests that the early postnatal period may represent an early developmental window whereby permanent alterations in DA function may lead to altered adult behavior.

### AN ALTERED DEVELOPMENTAL TRAJECTORY

#### Schizophrenia

One key facet of schizophrenia is the post-adolescent onset of “psychosis” or psychotic symptoms (Delisi, 1992). The wealth of epidemiological evidence for a neurodevelopmental pathogenesis as described throughout this review needs to be considered in the light of the post-adolescent onset of disease (Andreasen, 1995). The brain undergoes a high level of reorganization throughout adolescence (Spear, 2000; Andersen, 2003; Adriani and Laviola, 2004). Thus, the normal processes involved in brain maturation may be compromised such that the refinement of projections and signaling pathways uncovers or reveals an underlying dysfunction, such as altered genetic architecture (Lee et al., 2012) or subtle changes in neurochemical function (Howes et al., 2006; Howes et al., 2009).

The period of adolescent maturation is of particular importance to the clinical onset of psychotic disorders such as schizophrenia. For example, phencyclidine and ketamine (i.e., NMDA receptor antagonists) fail to produce hallucinations in prepubertal children, however they routinely do in adults (Hirsch et al., 1997). Thus the underlying connectivity/neurotransmission required for these drugs to elicit psychosis analogous to that seen in schizophrenia may not be functional until after adolescence. Obviously the presence of early onset schizophrenia suggests that these same processes *can* be present prior to adolescence but the fact remains that subtle alterations in cytoarchitecture, neurotransmission or brain connectivity may not yield a psychotic phenotype until these maturational processes are established. Importantly, the prodromal phase of schizophrenia that overlaps with this period of maturation is now being linked to dopaminergic abnormalities. For example, individuals at ultra-high risk of developing schizophrenia show increased striatal DA synthesis (Howes et al., 2006, 2009). There is also both behavioral and structural evidence that brain development is altered in people prior to disease onset. For example, behavioral abnormalities, IQ and social deficits have been described in children who later develop the disease (Aylward et al., 1984; Done et al., 1994; Ellenbroek and Cools, 1998; Murray and Fearon, 1999; Cannon et al., 2002, 2006). Moreover, at the onset of psychosis there are already changes in the gross anatomy of patients’ brains. For example, the lateral ventricles are increased in size (Chua et al., 2007; Nakamura et al., 2007; Pagsberg et al., 2007) and the cortex of schizophrenia patients frequently has decreased white and gray matter volume (Gur et al., 2000; Pantelis et al., 2003; Chua et al., 2007; Nakamura et al., 2007; Pagsberg et al., 2007). More recently two studies have shown that decreases in the gray matter volume of the parietal cortex and hippocampus precede the onset of psychosis in prodromal patients (Mechelli et al., 2011; Dazzan et al., 2012). Thus, there are a number of changes reflecting altered brain development and these appear to be present well before the onset of the symptoms required for clinical diagnosis.

Although cases of early onset schizophrenia suggest these adolescent maturational processes are not “key” per se, they still seem to play a significant role. For example, the decreases in cortical thickness found in early onset schizophrenia become more localized and more akin to those seen in adult onset schizophrenia when these patients reach adulthood (Greenstein et al., 2006) suggesting a role for adolescent maturation even in those with early onset schizophrenia. Furthermore, these examples also support a hierarchy of susceptibility in that highly susceptible individuals may be compromised earlier in life whereas others may require further extended environmental or developmental stressors to elicit frank symptoms. The fact that the enlarged lateral ventricles in DVD-deficient rats only persist to adulthood when the period of vitamin D deficiency is extended to weaning (Eyles et al., 2003; Feron et al., 2005) supports a titratable approach to brain susceptibility. In addition, it also suggests that further study on the temporal window of vitamin D deficiency would be extremely informative.

#### Rodent Analogs

There are notable similarities between adolescent/sexual maturation in rodents and humans. For example, the course of sexual maturation in rodents is preceded by the overproduction of synapses and accompanied by their subsequent elimination (Andersen et al., 1997). These dynamic changes in receptor density are thought to reflect the focussing and strengthening of synaptic connections required for adult life. This also occurs in humans with an estimated loss of almost one-half of the average number of synapses per cortical neuron over the adolescent period (Rakic et al., 1994). This period therefore represents a window whereby external influences prior to, rather than after, can differentially impact on adult brain function in both rodents and humans (Spear, 2000; Andersen, 2003; Adriani and Laviola, 2004). Of particular interest to both DVD deficiency and schizophrenia are the dynamic changes observed in the DA system over this period. For example, in the rat the density of both D1 and D2 receptors increase in the striatum prior to puberty, followed by their decline during puberty (Andersen et al., 1997). However, it is important to note that the development and maturation of the DA system is a dynamic process with behavioral and neurochemical responses continuing to change in rats for years after birth (Hebert and Gerhardt, 1999; Rutz et al., 2009).

Akin to drug sensitivities and the psychotic symptoms observed in schizophrenia, postpubertal psychomotor sensitivities to drugs such as amphetamine and MK-801 have been found in developmental animal models after DVD deficiency (Kesby et al., 2010), gestational disruptions in neurogenesis (Flagstad et al., 2004), neonatal ventral hippocampal lesions (Al-Amin et al., 2001) and prenatal Poly I:C administration (Meyer et al., 2008). Taken together these suggest a myriad of interventions can result in psychotic-like drug sensitivities that become observable after adolescence. Perhaps the most important aspect of these models is that they do not include any additional “stressor” during the adolescent period and are the sole results of early life interventions that alter normal brain development. Early intervention with antipsychotic treatment has already been shown to attenuate structural and behavioral abnormalities after prenatal Poly I:C administration (Piontkewitz et al., 2012) emphasizing that the developmental cascade prior to adolescence is critical to the “schizophrenia phenotype.” Furthermore, these models also provide the ability to investigate developmental abnormalities induced by a range of interventions and identify the convergent etiological pathways that result in similar adult behavioral phenotypes.

## DOPAMINE: A COMMON ENDPOINT OR A COMMON BEGINNING

**FIGURE 1:**
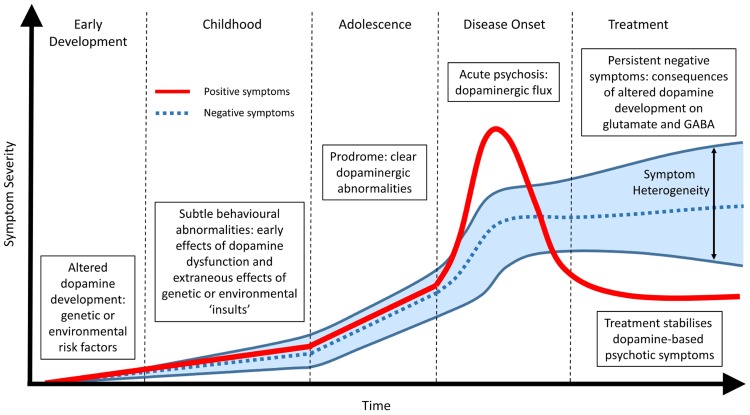
**Temporal profile of developing schizophrenia symptoms.** Early alterations in dopamine development due to genetic, environmental or a combination of both lead to abnormalities in dopamine function (positive symptoms) and subsequent alterations in other neurotransmitter systems (negative symptoms). During adolescence and the prodromal phase of the disease clear changes in dopamine function can be observed. Frank psychosis and disease onset are directly related to dopaminergic function and can be effectively treated. The lack of effect of antipsychotic treatment on persisting negative symptoms suggest they are not directly related to dopamine function. Rather, they represent the downstream consequence of early dopamine dysfunction or the extraneous effects of specific genetic and environmental risk factors on other neurotransmitter systems such as glutamate and GABA. These non-specific actions, outside of the core schizophrenia etiology, result in a large heterogeneous profile of negative symptoms in patients.

The premise that no single genetic vulnerability or molecular factor “causes” schizophrenia is well accepted amongst the research community and is confirmed by both the heterogeneity of the symptom profile and the lack of a diagnostic marker. A common endpoint, that includes aspects of DA dysfunction (Di Forti et al., 2007; Murray et al., 2008; Howes and Kapur, 2009), remains highly supported by the clinical evidence and allows for a specific outcome to investigate the etiology of the disease. However, we postulate that a common DA endpoint may arise precisely because it is central to the developmental pathology (**Figure 1**). The dopaminergic system is one of the most organized neurotransmitter systems in the brain and is fundamental for a range of functions involved in cognition, motivation and reward (Smith and Kieval, 2000; Aldridge et al., 2004; Nicola, 2007). Furthermore, alterations in DA signaling have a range of cascading effects on other neurotransmitter systems such as glutamate (e.g., NMDA) and GABA. Therefore, even small alterations in DA function or organization have the potential to lead to complex cognitive outcomes when coupled with other general and variable insults such as altered genetic architecture, drug use, stress or adolescent maturation. In addition, these secondary stressors may individually produce differing phenotypes and thus heterogeneity in symptom profile.

Our work the in DVD-deficient rat model suggests that the developmental absence of this ligand produces discrete alterations in how DA systems develop. Alterations in DA specification factors (Cui et al., 2010) and DA metabolism (Kesby et al., 2009) induced by DVD deficiency could have lasting influences on the DA signaling. Additionally, these animals show behavioral sensitivities to psychomimetic drugs at adulthood (Kesby et al., 2006, 2010, 2011; O’Loan et al., 2007) which model at least the positive symptoms of schizophrenia (Laruelle and Abi-Dargham, 1999; Lahti et al., 2001). Moreover, another developmental model, that utilizes PolyI:C to mimic prenatal infection, also shows alterations in Nurr 1, and similar behavioral sensitivities to these psychomimetic drugs in adulthood (Meyer et al., 2008; Vuillermot et al., 2010). Taken together this suggests that vastly differing developmental insults can cause similar phenotypes and perhaps even converge on common early mechanisms (Eyles et al., 2012). Thus a network of small communication errors, perhaps via the interaction of a variety of key receptors (RXR, VDR, Nurr1, Ret) result in an altered developmental landscape. This may lead to an altered number of DA neurons after perinatal cell death events or altered DA functionality/connectivity.

The data amassed in the DVD-deficient rat suggest that subtle developmental alterations in DA function can lead to an altered adult behavioral phenotype. Moreover, utilizing differing developmental insults, animal models have demonstrated similar developmental dopaminergic abnormalities and adult behavioral phenotypes. Therefore, schizophrenia may be a disorder occurring via genetic or environmental factors that features a common early disruption in DA development. The emerging data suggesting that altered DA function precedes the onset of psychosis further suggests that dopaminergic abnormalities are not a byproduct of psychosis but rather a latent signal of altered DA development. Thus, altered dopaminergic function in schizophrenia may represent a potential biological marker and even a target for intervention.

## CONCLUSION

Schizophrenia is an extremely complex disorder. We are not suggesting that future therapeutic interventions be limited to a simple “fix DA early and fix schizophrenia” interpretation. However, observations in the DVD-deficient rat, alternative animal models and existing clinical evidence suggests that a core/common feature may be early DA dysfunction. That this could originate from an early alteration in DA development is not a radical premise but the concept itself is extremely hard to assess in humans for a variety of obvious temporal and ethical reasons. However, understanding how the potential cascade of events after early alterations in DA neuron development influence other neurotransmitter systems in animal models, such as the DVD-deficient rat, may increase our understanding of the actual etiology of schizophrenia. Furthermore, subtle alterations to DA development may provide a platform to learn and understand the variable outcomes associated with a range of “second-hit” targets. The future of basic schizophrenia research should be focused on early dopaminergic development with the goal of further understanding existing and underlying abnormalities in schizophrenia patients that may, in turn, direct treatment solutions in the clinic.

## Conflict of Interest Statement

The authors declare that the research was conducted in the absence of any commercial or financial relationships that could be construed as a potential conflict of interest.

## References

[B1] Aarnisalo P. Kim C. H. Lee J. W. Perlmann T. (2002). Defining requirements for heterodimerization between the retinoid X receptor and the orphan nuclear receptor Nurr1. *J. Biol. Chem.* 277 35118–35123 10.1074/jbc.M201707200 12130634

[B2] Abi-Dargham A. Gil R. Krystal J. Baldwin R. M. Seibyl J. P. Bowers M. (1998). Increased striatal dopamine transmission in schizophrenia: confirmation in a second cohort. *Am. J. Psychiat.* 155 761–767 9619147 10.1176/ajp.155.6.761

[B3] Abi-Dargham A. van de Giessen E. Slifstein M. Kegeles L. S. Laruelle M. (2009). Baseline and amphetamine-stimulated dopamine activity are related in drug-naive schizophrenic subjects. *Biol. Psychiatry* 65 1091–1093 10.1016/j.biopsych.2008.12.007 19167701

[B4] Adriani W. Laviola G. (2004). Windows of vulnerability to psychopathology and therapeutic strategy in the adolescent rodent model. *Behav. Pharmacol.* 15 341–352 10.1097/00008877-200409000-00005 15343057

[B5] Al-Amin H. A. Weickert C. S. Weinberger D. R. Lipska B. K. (2001). Delayed onset of enhanced MK-801-induced motor hyperactivity after neonatal lesions of the rat ventral hippocampus. *Biol. Psychiatry* 49 528–539 10.1016/S0006-3223(00)00968-9 11257238

[B6] Aldridge J. W. Berridge K. C. Rosen A. R. (2004). Basal ganglia neural mechanisms of natural movement sequences. *Can. J. Physiol. Pharmacol.* 82 732–739 10.1139/y04-061 15523530

[B7] Andersen S. L. (2003). Trajectories of brain development: point of vulnerability or window of opportunity? *Neurosci. Biobehav. Rev*. 27 3–18 10.1016/S0149-7634(03)00005-8 12732219

[B8] Andersen S. L. Rutstein M. Benzo J. M. Hostetter J. C. Teicher M. H. (1997). Sex differences in dopamine receptor overproduction and elimination. *Neuroreport* 8 1495–1498 10.1097/00001756-199704140-00034 9172161

[B9] Andine P. Widermark N. Axelsson R. Nyberg G. Olofsson U. Martensson E. (1999). Characterization of MK-801-induced behavior as a putative rat model of psychosis. *J. Pharmacol. Exp. Ther.* 290 1393–1408 10454519

[B10] Andreasen N. C. (1995). Symptoms, signs, and diagnosis of schizophrenia. *Lancet* 346 477–481 10.1016/S0140-6736(95)91325-4 7637483

[B11] Angrist B. Vankammen D. P. (1984). Cns stimulants as tools in the study of schizophrenia. *Trends Neurosci.* 7 388–390 10.1016/S0166-2236(84)80062-4

[B12] Aylward E. Walker E. Bettes B. (1984). Intelligence in schizophrenia: meta-analysis of the research. *Schizophr. Bull.* 10 430–459 10.1093/schbul/10.3.430 6382590

[B13] Banerjee P. Chatterjee M. (2003). Antiproliferative role of vitamin D and its analogs – a brief overview. *Mol. Cell. Biochem.* 253 247–254 10.1023/A:1026072118217 14619976

[B14] Becker A. Eyles D. W. McGrath J. J. Grecksch G. (2005). Transient prenatal vitamin D deficiency is associated with subtle alterations in learning and memory functions in adult rats. *Behav. Brain Res.* 161 306–312 10.1016/j.bbr.2005.02.015 15922058

[B15] Bernert G. Hoeger H. Mosgoeller W. Stolzlechner D. Lubec B. (2003). Neurodegeneration, neuronal loss, and neurotransmitter changes in the adult guinea pig with perinatal asphyxia. *Pediatr. Res.* 54 523–528 10.1203/01.PDR.0000081760.48331.7A 12867599

[B16] Bjelke B. Andersson K. Ogren S. O. Bolme P. (1991). Asphyctic Lesion – Proliferation of tyrosine hydroxylase-immunoreactive nerve-cell bodies in the rat substantia-nigra and functional-changes in dopamine neurotransmission. *Brain Res.* 543 1–9 10.1016/0006-8993(91)91041-X 1675922

[B17] Boksa P. El-Khodor B. F. (2003). Birth insult interacts with stress at adulthood to alter dopaminergic function in animal models: possible implications for schizophrenia and other disorders. *Neurosci. Biobehav. Rev.* 27 91–101 10.1016/S0149-7634(03)00012-5 12732226

[B18] Breier A. Su T. P. Saunders R. Carson R. E. Kolachana B. S. deBartolomeis A. (1997). Schizophrenia is associated with elevated amphetamine-induced synaptic dopamine concentrations: evidence from a novel positron emission tomography method. *Proc. Natl. Acad. Sci. U.S.A.* 94 2569–2574 10.1073/pnas.94.6.2569 9122236 PMC20129

[B19] Burke R. E. (2004). Ontogenic cell death in the nigrostriatal system. *Cell Tissue Res.* 318 63–72 10.1007/s00441-004-0908-4 15349767

[B20] Burket R. McGrath J. Eyles D. (2003). Vitamin D receptor expression in the embryonic rat brain. *Neurosci. Res. Commun.* 33 63–71 10.1002/nrc.10081

[B21] Burne T. H. J. Becker A. Brown J. Eyles D. W. Mackay-Sim A. McGrath J. J. (2004). Transient prenatal Vitamin D deficiency is associated with hyperlocomotion in adult rats. *Behav. Brain Res.* 154 549–555 10.1016/j.bbr.2004.03.023 15313044

[B22] Burne T. H. J. O’Loan J. McGrath J. J. Eyles D. W. (2006). Hyperlocomotion associated with transient prenatal vitamin D deficiency is ameliorated by acute restraint. *Behav. Brain Res.* 174 119–124 10.1016/j.bbr.2006.07.015 16930734

[B23] Burne T. H. J. O’Loan J. Splatt K. Alexander S. McGrath J. J. Eyles D. W. (2011). Developmental vitamin D (DVD) deficiency alters pup-retrieval but not isolation-induced pup ultrasonic vocalizations in the rat. *Physiol. Behav.* 102 201–204 10.1016/j.physbeh.2010.11.006 21059363

[B24] Cannon M. Caspi A. Moffitt T. E. Harrington H. Taylor A. Murray R. M. (2002). Evidence for early-childhood, pan-developmental impairment specific to schizophreniform disorder – results from a longitudinal birth cohort. *Arch. Gen. Psychiatry* 59 449–456 10.1001/archpsyc.59.5.449 11982449

[B25] Cannon M. Moffitt T. E. Caspi A. Murray R. M. Harrington H. Poulton R. (2006). Neuropsychological performance at the age of 13 years and adult schizophreniform disorder – prospective birth cohort study. *Br. J. Psychiatry* 189 463–464 10.1192/bjp.bp.105.020552 17077440

[B26] Cantor-Graae E. Selten J. P. (2005). Schizophrenia and migration: a meta-analysis and review. *Am. J. Psychiatry* 162 12–24 10.1176/appi.ajp.162.1.12 15625195

[B27] Carlsson A. Lindqvist M. (1963). Effect of chlorpromazine or haloperidol on formation of 3methoxytyramine and normetanephrine in mouse brain. *Acta Pharmacol. Toxicol. (Copenh)* 20 140–144 10.1111/j.1600-0773.1963.tb01730.x 14060771

[B28] Carlsson M. Carlsson A. (1989). The NMDA antagonist MK-801 causes marked locomotor stimulation in monoamine-depleted mice. *J. Neural Transm.* 75 221–226 10.1007/BF01258633 2538557

[B29] Chefer V. I. Zakharova I. Shippenberg T. S. (2003). Enhanced responsiveness to novelty and cocaine is associated with decreased basal dopamine uptake and release in the nucleus accumbens: quantitative microdialysis in rats under transient conditions. *J. Neurosci.* 23 3076–3084 12684494 10.1523/JNEUROSCI.23-07-03076.2003 PMC6742067

[B30] Christakos S. Dhawan P. Liu Y. Peng X. R. Porta A. (2003). New insights into the mechanisms of vitamin D action. *J. Cell. Biochem.* 88 695–705 10.1002/jcb.10423 12577303

[B31] Chua S. E. Cheung C. Cheung V. Tsang J. T. K. Chen E. Y. H. Wong J. C. H. (2007). Cerebral grey, white matter and csf in never-medicated, first-episode schizophrenia. *Schizophr. Res.* 89 12–21 10.1016/j.schres.2006.09.009 17098398

[B32] Chun H. S. Yoo M. S. DeGiorgio L. A. Volpe B. T. Peng D. Baker H. (2002). Marked dopaminergic cell loss subsequent to developmental, intranigral expression of glial cell line-derived neurotrophic factor. *Exp. Neurol.* 173 235–244 10.1006/exnr.2001.7842 11822887

[B33] Clemens T. L. Adams J. S. Henderson S. L. Holick M. F. (1982). Increased skin pigment reduces the capacity of skin to synthesise vitamin D3. *Lancet* 1 74–76 10.1016/S0140-6736(82)90214-8 6119494

[B34] Clemens T. L. Garrett K. P. Zhou X. Y. Pike J. W. Haussler M. R. Dempster D. W. (1988). Immunocytochemical localization of the 1,25-dihydroxyvitamin D3 receptor in target cells. *Endocrinology* 122 1224–1230 10.1210/endo-122-4-1224 2831024

[B35] Costa A. la Fougere C. Pogarell O. Moller H. J. Riedel M. Ettinger U. (2012). Impulsivity is related to striatal dopamine transporter availability in healthy males. *Psychiatry Res*. 211 251–256 10.1016/j.pscychresns.2012.07.011 23158972

[B36] Creese I. Burt D. R. Snyder S. H. (1976). Dopamine receptor binding predicts clinical and pharmacological potencies of antischizophrenic drugs. *Science* 192 481–483 10.1126/science.3854 3854

[B37] Criswell H. E. Johnson K. B. Mueller R. A. Breese G. R. (1993). Evidence for involvement of brain dopamine and other mechanisms in the behavioral action of the *N*-Methyl-D-aspartic acid antagonist Mk-801 in control and 6-hydroxydopamine-lesioned rats. *J. Pharmacol. Exp. Ther.* 265 1001–1010 8098756

[B38] Cui X. Pelekanos M. Liu P.-Y. Burne T. H. J. McGrath J. J. Eyles D. W. (2013). The vitamin D receptor in dopamine neurons; its presence in human substantia nigra and its ontogenesis in rat midbrain. *Neuroscience* 236 77–87 10.1016/j.neuroscience.2013.01.035 23352937

[B39] Cui X. Y. McGrath J. J. Burne T. H. J. Mackay-Sim A. Eyles D. W. (2007). Maternal vitamin D depletion alters neurogenesis in the developing rat brain. *Int. J. Dev. Neurosci.* 25 227–232 10.1016/j.ijdevneu.2007.03.006 17467223

[B40] Cui X. Y. Pelekanos M. Burne T. H. J. McGrath J. J. Eyles D. W. (2010). Maternal vitamin D deficiency alters the expression of genes involved in dopamine specification in the developing rat mesencephalon. *Neurosci. Lett.* 486 220–223 10.1016/j.neulet.2010.09.057 20884326

[B41] Davies G. Welham J. Chant D. Torrey E. F. McGrath J. (2003). A systematic review and meta-analysis of Northern Hemisphere season of birth studies in schizophrenia. *Schizophr. Bull.* 29 587–593 10.1093/oxfordjournals.schbul.a007030 14609251

[B42] Davis K. L. Kahn R. S. Ko G. Davidson M. (1991). Dopamine in schizophrenia – a review and reconceptualization. *Am. J. Psychiatry* 148 1474–1486 1681750 10.1176/ajp.148.11.1474

[B43] Dazzan P. Soulsby B. Mechelli A. Wood S. J. Velakoulis D. Phillips L. J. (2012). Volumetric abnormalities predating the onset of schizophrenia and affective psychoses: an MRI study in subjects at ultrahigh risk of psychosis. *Schizophr. Bull.* 38 1083–1091 10.1093/schbul/sbr035 21518921 PMC3446227

[B44] Decker M. J. Hue G. E. Caudle W. M. Miller G. W. Keating G. L. Rye D. B. (2003). Episodic neonatal hypoxia evokes executive dysfunction and regionally specific alterations in markers of dopamine signaling. *Neuroscience* 117 417–425 10.1016/S0306-4522(02)00805-9 12614682

[B45] Decressac M. Kadkhodaei B. Mattsson B. Laguna A. Perlmann T. Bjorklund A. (2012). α -synuclein-induced down-regulation of Nurr1 disrupts GDNF signaling in nigral dopamine neurons. *Sci. Transl. Med.* 4 63ra156 10.1126/scitranslmed.3004676 23220632

[B46] Delisi L. E. (1992). The significance of age of onset for schizophrenia. *Schizophr. Bull.* 18 209–215 10.1093/schbul/18.2.209 1377833

[B47] Di Forti M. Lappin J. M. Murray R. M. (2007). Risk factors for schizophrenia – all roads lead to dopamine. *Eur. Neuropsychopharmology* 17 S101–S107 10.1016/j.euroneuro.2007.02.005 17336764

[B48] Done D. J. Crow T. J. Johnstone E. C. Sacker A. (1994). Childhood antecedents of schizophrenia and affective illness: social adjustment at ages 7 and 11. *BMJ* 309 699–703 10.1136/bmj.309.6956.699 7950522 PMC2540822

[B49] Dusso A. S. Brown A. J. Slatopolsky E. (2005). Vitamin D. *Am. J. Physiol. Renal Physiol.* 289 F8–F28 10.1152/ajprenal.00336.2004 15951480

[B50] Ellenbroek B. A. Cools A. R. (1998). The neurodevelopment hypothesis of schizophrenia: clinical evidence and animal models. *Neurosci. Res. Commun.* 22 127–136 10.1002/(SICI)1520-6769(199805/06)22

[B51] Erben R. G. Soegiarto D. W. Weber K. Zeitz U. Lieberherr M. Gniadecki R. (2002). Deletion of deoxyribonucleic acid binding domain of the vitamin D receptor abrogates genomic and nongenomic functions of vitamin D. *Mol. Endocrinol.* 16 1524–1537 10.1210/me.16.7.1524 12089348

[B52] Eyles D. Anderson C. Ko P. Jones A. Thomas A. Burne T. (2009). A sensitive LC/MS/MS assay of 250H vitamin D-3 and 250H vitamin D-2 in dried blood spots. *Clin. Chim. Acta* 403 145–151 10.1016/j.cca.2009.02.005 19232332

[B53] Eyles D. Brown J. Mackay-Sim A. McGrath J. Feron F. (2003). Vitamin D-3 and brain development. *Neuroscience* 118 641–653 10.1016/S0306-4522(03)00040-X 12710973

[B54] Eyles D. Feldon J. Meyer U. (2012). Schizophrenia: do all roads lead to dopamine or is this where they start? Evidence from two epidemiologically informed developmental rodent models. *Transl. Psychiatry* 2 e81 10.1038/tp.2012.6 PMC3309552 22832818

[B55] Eyles D. W. Smith S. Kinobe R. Hewison M. McGrath J. J. (2005). Distribution of the vitamin D receptor and 1 alpha-hydroxylase in human brain. *J. Chem. Neuroanat.* 29 21–30 10.1016/j.jchemneu.2004.08.006 15589699

[B56] Eyles D. W. Rogers F. Buller K. McGrath J. J. Ko P. French K. (2006). Developmental vitamin D (DVD) deficiency in the rat alters adult behaviour independently of HPA function. *Psychoneuroendocrinology* 31 958–964 10.1016/j.psyneuen.2006.05.006 16890375

[B57] Feenstra M. G. P. Botterblom M. H. A Vanuum J. F. M. (1995). Novelty-induced increase in dopamine release in the rat prefrontal cortex in-vivo – inhibition by diazepam. *Neurosci. Lett.* 189 81–84 10.1016/0304-3940(95)11456-7 7609924

[B58] Feron F. Burne T. H. J. Brown J. Smith E. McGrath J. J. Mackay-Sim A. (2005). Developmental Vitamin D-3 deficiency alters the adult rat brain. *Brain Res. Bull.* 65 141–148 10.1016/j.brainresbull.2004.12.007 15763180

[B59] Flagstad P. Mork A. Glenthoj B. Y. van Beek J. Michael-Titus A. T. Didriksen M. (2004). Disruption of neurogenesis on gestational day 17 in the rat causes behavioral changes relevant to positive and negative schizophrenia symptoms and alters amphetamine-induced dopamine release in nucleus accumbens. *Neuropsychopharmacology* 29 2052–2064 10.1038/sj.npp.1300516 15199377

[B60] Fu G. K. Lin D. Zhang M. Y. H. Bikle D. D. Shackleton C. H. L. Miller W. L. (1997). Cloning of human 25-hydroxyvitamin D-1 alpha-hydroxylase and mutations causing vitamin D-dependent rickets type 1. *Mol. Endocrinol.* 11 1961–1970 10.1210/me.11.13.1961 9415400

[B61] Fusar-Poli P. Meyer-Lindenberg A. (2013). Striatal presynaptic dopamine in schizophrenia, Part II: meta-analysis of F-18/C-11 -DOPA PET studies. *Schizophr. Bull.* 39 33–42 10.1093/schbul/sbr180 22282454 PMC3523905

[B62] Gates M. A. Torres E. M. White A. Fricker-Gates R. A. Dunnett S. B. (2006). Re-examining the ontogeny of substantia nigra dopamine neurons. *Eur. J. Neurosci.* 23 1384–1390 10.1111/j.1460-9568.2006.04637.x 16553799

[B63] Gezen-Ak D. Dursun E. Yilmazer S. (2011). The effects of vitamin D receptor silencing on the expression of LVSCC-A1C and LVSCC-A1D and the release of NGF in cortical neurons. *PLoS ONE * 6:e17553 10.1371/journal.pone.0017553 PMC3048291 21408608

[B64] Gray J. A. Feldon J. Rawlins J. N. P. Smith A. D. Hemsley D. R. (1991). The neuropsychology of schizophrenia. *Behav. Brain Sci.* 14 1–19 10.1017/S0140525X00065055

[B65] Greenstein D. Lerch J. Shaw P. Clasen L. Giedd J. Gochman P. (2006). Childhood onset schizophrenia: cortical brain abnormalities as young adults. *J. Child Psychol. Psychiatry* 47 1003–1012 10.1111/j.1469-7610.2006.01658.x 17073979

[B66] Gur R. E. Turetsky B. I. Cowell P. E. Finkelman C. Maany V. Grossman R. I. (2000). Temporolimbic volume reductions in schizophrenia. *Arch. Gen. Psychiatry* 57 769–775 10.1001/archpsyc.57.8.769 10920465

[B67] Hebert M. A. Gerhardt G. A. (1999). Age-related changes in the capacity, rate, and modulation of dopamine uptake within the striatum and nucleus accumbens of Fischer 344 rats: an in vivo electrochemical study. *J. Pharmacol. Exp. Ther.* 288 879–887 9918602

[B68] Hirsch S. R. Das I. Garey L. J. deBelleroche J. (1997). A pivotal role for glutamate in the pathogenesis of schizophrenia, and its cognitive dysfunction. *Pharmacol. Biochem. Behav.* 56 797–802 10.1016/S0091-3057(96)00428-5 9130307

[B69] Holick M. F. Matsuoka L. Y. Wortsman J. (1995). Regular use of sunscreen on vitamin D levels. *Arch. Dermatol.* 131 1337–1339 10.1001/archderm.131.11.1337 7503584

[B70] Hooks M. S. Kalivas P. W. (1995). The Role of mesoaccumbens pallidal circuitry in novelty-induced behavioral activation. *Neuroscience* 64 587–597 10.1016/0306-4522(94)00409-X 7715773

[B71] Howes O. Bose S. Turkheimer F. Valli I. Egerton A. Stahl D. (2011a). Progressive increase in striatal dopamine synthesis capacity as patients develop psychosis: a PET study. *Mol. Psychiatry* 16 885–886 10.1038/mp.2011.20 21358709 PMC3662873

[B72] Howes O. D. Kapur S. (2009). The dopamine hypothesis of schizophrenia: version III-025EFThe final common pathway. *Schizophr. Bull.* 35 549–562 10.1093/schbul/sbp006 19325164 PMC2669582

[B73] Howes O. D. Montgomery A. J. Asselin M. C. Murray R. M. Grasby P. M. McGuire P. K. (2006). The pre-synaptic dopaminergic system before and after the onset of psychosis: initial results. *Eur. Neuropsychopharmacol*. 16 S177 10.1016/S0924-977X(06)70050-5

[B74] Howes O. D. Kambeitz J. Kim E. Stahl D. Slifstein M. Abi-Dargham A. (2012). The nature of dopamine dysfunction in schizophrenia and what this means for treatment. *Arch. Gen. Psychiatry* 69 776–786 10.1001/archgenpsychiatry.2012.169 22474070 PMC3730746

[B75] Howes O. D. Bose S. K. Turkheimer F. Valli I. Egerton A. Valmaggia L. R. (2011b). Dopamine synthesis capacity before onset of psychosis: a prospective (18)F -DOPA PET Imaging Study. *Am. J.. Psychiatry* 168 1311–1317 10.1176/appi.ajp.2011.11010160 21768612 PMC3682447

[B76] Howes O. D. Montgomery A. J. Asselin M. C. Murray R. M. Valli I. Tabraham P. (2009). Elevated striatal dopamine function linked to prodromal signs of schizophrenia. *Arch. Gen. Psychiatry* 66 13–20 10.1001/archgenpsychiatry.2008.514 19124684

[B77] Iwawaki T. Kohno K. Kobayashi K. (2000). Identification of a potential Nurr1 response element that activates the tyrosine hydroxylase gene promoter in cultured cells. *Biochem. Biophys. Res. Commun.* 274 590–595 10.1006/bbrc.2000.3204 10924322

[B78] Janowsky D. S. Elyousef M. K. Davis J. M. Sekerke H. J. (1973). Provocation of schizophrenic symptoms by intravenous administration of methylphenidate. *Arch. Gen. Psychiatry* 28 185–191 10.1001/archpsyc.1973.01750320023004 4630714

[B79] Jones S. R. Gainetdinov R. R. Wightman R. M. Caron M. G. (1998). Mechanisms of amphetamine action revealed in mice lacking the dopamine transporter. *J. Neurosci.* 18 1979–1986 9482784 10.1523/JNEUROSCI.18-06-01979.1998 PMC6792915

[B80] Joseph B. Wallen-Mackenzie A. Benoit G. Murata T. Joodmardi E. Okret S. (2003). p57(Kip2) cooperates with Nurr1 in developing dopamine cells. *Proc. Natl. Acad. Sci. U.S.A.* 100 15619–15624 10.1073/pnas.2635658100 14671317 PMC307617

[B81] Kellendonk C. Simpson E. H. Polan H. J. Malleret G. Vronskaya S. Winiger V. (2006). Transient and selective overexpression of dopamine D2 receptors in the striatum causes persistent abnormalities in prefrontal cortex functioning. *Neuron* 49 603–615 10.1016/j.neuron.2006.01.023 16476668

[B82] Kesby J. P. Burne T. H. J. McGrath J. J. Eyles D. W. (2006). Developmental vitamin D deficiency alters MK 801-induced hyperlocomotion in the adult rat: an animal model of schizophrenia. *Biol. Psychiatry* 60 591–596 10.1016/j.biopsych.2006.02.033 16697353

[B83] Kesby J. P. Cui X. Ko P. McGrath J. J. Burne T. H. J. Eyles D. W. (2009). Developmental vitamin D deficiency alters dopamine turnover in neonatal rat forebrain. *Neurosci. Lett.* 461 155–158 10.1016/j.neulet.2009.05.070 19500655

[B84] Kesby J. P. Cui X. O’Loan J. McGrath J. J. Burne T. H. J. Eyles D. W. (2010). Developmental vitamin D deficiency alters dopamine-mediated behaviors and dopamine transporter function in adult female rats. *Psychopharmacology* 159–168 10.1007/s00213-009-1717-y 19921153

[B85] Kesby J. P. O’Loan J. C. Alexander S. Deng C. Huang X. F. McGrath J. J. (2011). Developmental vitamin D deficiency alters MK-801-induced behaviours in adult offspring. *Psychopharmacology (Berl.)* 220 455–463 10.1007/s00213-011-2492-0 21947313

[B86] Kholodilov N. Yarygina O. Oo T. F. Zhang H. Sulzer D. Dauer W. (2004). Regulation of the development of mesencephalic dopaminergic systems by the selective expression of glial cell line-derived neurotrophic factor in their targets. *J. Neurosci.* 24 3136–3146 10.1523/JNEUROSCI.4506-03.2004 15044553 PMC6729846

[B87] Kim K. S. Kim C. H. Hwang D. Y. Seo H. Chung S. M. Hong S. J. (2003). Orphan nuclear receptor Nurr1 directly transactivates the promoter activity of the tyrosine hydroxylase gene in a cell-specific manner. *J. Neurochem.* 85 622–634 10.1046/j.1471-4159.2003.01671.x 12694388

[B88] Ko P. Burkert R. McGrath J. Eyles D. (2004). Matemal vitamin D-3 deprivation and the regulation of apoptosis and cell cycle during rat brain development. *Brain Res. Dev. Brain Res.* 153 61–68 10.1016/j.devbrainres.2004.07.013 15464218

[B89] Krystal J. H. Karper L. P. Seibyl J. P. Freeman G. K. Delaney R. Bremner J. D. (1994). Subanesthetic effects of the noncompetitive NMDA antagonist, ketamine, in humans. Psychotomimetic, perceptual, cognitive, and neuroendocrine responses. *Arch. Gen.Psychiatry* 51 199–214 10.1001/archpsyc.1994.03950030035004 8122957

[B90] Lahti A. C. Weiler M. A. Tamara Michaelidis B. A. Parwani A. Tamminga C. A. (2001). Effects of ketamine in normal and schizophrenic volunteers. *Neuropsychopharmacology* 25 455–467 10.1016/S0893-133X(01)00243-3 11557159

[B91] Laruelle M. (1998). Imaging dopamine transmission in schizophrenia. A review and meta-analysis*. Q. J. Nucl. Med.* 42 211–221 9796369

[B92] Laruelle M. Abi-Dargham P. (1999). Dopamine as the wind of the psychotic fire: new evidence from brain imaging studies. *J. Psychopharmacol.* 13 358–371 10.1177/026988119901300405 10667612

[B93] Laruelle M. Abi-Dargham A. Gil R. Kegeles L. Innis R. (1999). Increased dopamine transmission in schizophrenia: relationship to illness phases. *Biol. Psychiatry * 46 56–72 10.1016/S0006-3223(99)00067-0 10394474

[B94] Laruelle M. Abi-Dargham A. van Dyck C. H. Gil R. D’Souza C. D. Erdos J. (1996). Single photon emission computerized tomography imaging of amphetamine-induced dopamine release in drug-free schizophrenic subjects. *Proc. Natl. Acad. Sci. U.S.A.* 93 9235–9240 10.1073/pnas.93.17.9235 8799184 PMC38625

[B95] Lauder J. M. Bloom F. E. (1974). Ontogeny of monoamine neurons in locus coeruleus, raphe nuclei and substantia nigra of rat.1. Cell-Differentiation. * J. Comp. Neurol.* 155 469–481 10.1002/cne.901550407 4847734

[B96] Lee S. H. DeCandia T. R. Ripke S. Yang J. Sullivan P. F. Goddard M. E. (2012). Estimating the proportion of variation in susceptibility to schizophrenia captured by common SNPs. *Nat. Genet.* 44 U235–U247 10.1038/ng.1108 PMC3327879 22344220

[B97] Li Y. M. Cong B. Ma C. L. Qi Q. A. Fu L. H. Zhang G. Z. (2011). Expression of Nurr1 during rat brain and spinal cord development. *Neurosci. Lett.* 488 49–54 10.1016/j.neulet.2010.10.078 21056632

[B98] Lieberman J. A. Kane J. M. Alvir J. (1987). Provocative tests with psychostimulant drugs in schizophrenia. *Psychopharmacology (Berl.)* 91 415–433 10.1007/BF00216006 2884687

[B99] Lin L. F. H. Doherty D. H. Lile J. D. Bektesh S. Collins F. (1993). Gdnf – a glial-cell line derived neurotrophic factor for midbrain dopaminergic-neurons. *Science* 260 1130–1132 10.1126/science.8493557 8493557

[B100] Lubow R. E. Gewirtz J. C. (1995). Latent inhibition in humans – data, theory, and implications for schizophrenia. *Psychol. Bull.* 117 87–103 10.1037/0033-2909.117.1.87 7870865

[B101] Mangelsdorf D. J. Thummel C. Beato M. Herrlich P. Schutz G. Umesono K. (1995). The nuclear receptor superfamily – the 2nd decade. *Cell* 83 835–839 10.1016/0092-8674(95)90199-X 8521507 PMC6159888

[B102] McGrath J. (1999). Hypotheses: is low prenatal vitamin D a risk-modifying factor for schizophrenia? *Schizophr. Res*. 40 173–177 10.1016/S0920-9964(99)00052-3 10638855

[B103] McGrath J. J. Kimlin M. G. Saha S. Eyles D. W. Parisi A. V. (2001). Vitamin D insufficiency in south-east Queensland. *Med. J. Aust.* 174 150–151 11247622 10.5694/j.1326-5377.2001.tb143195.x

[B104] McGrath J. J. Saari K. Hakko H. Jokelainen J. Jones P. B. Jarvelin M. R. (2004). Vitamin D supplementation during the first year of life and risk of schizophrenia: a Finnish birth-cohort study. *Schizophr. Res.* 67 16–16 10.1016/j.schres.2003.08.005 14984883

[B105] McGrath J. J. Eyles D. W. Pedersen C. B. Anderson C. Ko P. Burne T. H. (2010). Neonatal vitamin D status and risk of schizophrenia: a population-based case-control study. *Arch. Gen. Psychiatry* 67 889–894 10.1001/archgenpsychiatry.2010.110 20819982

[B106] Mechelli A. Riecher-Rossler A. Meisenzahl E. M. Tognin S. Wood S. J. Borgwardt S. J. (2011). Neuroanatomical abnormalities that predate the onset of psychosis a multicenter study. *Arch. Gen. Psychiatry* 68 489–495 10.1001/archgenpsychiatry.2011.42 21536978

[B107] Meyer U. Feldon J. Fatemi S. H. (2009) In-vivo rodent models for the experimental investigation of prenatal immune activation effects in neurodevelopmental brain disorders. *Neurosci .Biobehav. Rev*. 33 1061–1079 10.1016/j.neubiorev.2009.05.001 19442688

[B108] Meyer U. Nyffeler M. Schwendener S. Knuesel I. Yee B. K. Feldon J. (2008). Relative prenatal and postnatal maternal contributions to schizophrenia-related neurochemical dysfunction after in utero immune challenge. *Neuropsychopharmacology* 33 441–456 10.1038/sj.npp.1301413 17443130

[B109] Murray R. M. Lewis S. W. (1987). Is schizophrenia a neurodevelopmental disorder. *Br. Med. J.* 295 681–682 10.1136/bmj.295.6600.681 3117295 PMC1247717

[B110] Murray R. M. Fearon P. (1999). The developmental ‘risk factor’ model of schizophrenia. *J. Psychiatr. Res.* 33 497–499 10.1016/S0022-3956(99)00032-1 10628525

[B111] Murray R. M. Lappin J Di Forti M. (2008). Schizophrenia: from developmental deviance to dopamine dysregulation. *Eur. Neuropsychopharmacol.* 18 S129–S134 10.1016/j.euroneuro.2008.04.002 18499406

[B112] Nakamura M. Salisbury D. F. Hirayasu Y. Bouix S. Pohl K. M. Yoshida T. (2007). Neocortical gray matter volume in first-episode schizophrenia and first-episode affective psychosis: a cross-sectional and longitudinal MRI study. *Biol. Psychiatry* 62 773–783 10.1016/j.biopsych.2007.03.030 17586477 PMC2782514

[B113] Nicola S. M. (2007). The nucleus accumbens as part of a basal ganglia action selection circuit. *Psychopharmacology (Berl.)* 191 521–550 10.1007/s00213-006-0510-4 16983543

[B114] Nomura Y. Yotsumoto I. Segawa T. (1981). Ontogenetic development of high potassium-induced and acetylcholine-induced release of dopamine from striatal slices of the rat. *Dev. Brain Res.* 1 171–177 10.1016/0165-3806(81)90105-X 7225887

[B115] O’Loan J. Eyles D. W. Kesby J. Ko P. McGrath J. J Burne T. H. J. (2007). Vitamin D deficiency during various stages of pregnancy in the rat; its impact on development and behaviour in adult offspring. *Psychoneuroendocrinology* 32 227–234 10.1016/j.psyneuen.2006.12.006 17276604

[B116] Olney J. W. Farber N. B. (1995). Glutamate receptor dysfunction and schizophrenia. *Arch. Gen. Psychiatry* 52 998–1007 10.1001/archpsyc.1995.03950240016004 7492260

[B117] Oo T. F. Burke R. E. (1997). The time course of developmental cell death in phenotypically defined dopaminergic neurons of the substantia nigra. *Dev. Brain Res.* 98 191–196 10.1016/S0165-3806(96)00173-3 9051260

[B118] Oo T. F. Kholodilov N. Burke R. E. (2003). Regulation of natural cell death in dopaminergic neurons of the substantia nigra by striatal glial cell line-derived neurotrophic factor in vivo. *J. Neurosci.* 23 5141–5148 12832538 10.1523/JNEUROSCI.23-12-05141.2003 PMC6741204

[B119] Orme R. P. Bhangal M. S. Fricker R. A. (2013) Calcitriol imparts neuroprotection in vitro to midbrain dopaminergic neurons by upregulating GDNF expression. *PLoS ONE* 8:e62040 10.1371/journal.pone.0062040 PMC3633905 23626767

[B120] Ozawa K. Hashimoto K. Kishimoto T. Shimizu E. Ishikura H. Iyo M. (2006). Immune activation during pregnancy in mice leads to dopaminergic hyperfunction and cognitive impairment in the offspring: a neurodevelopmental animal model of schizophrenia. *Biol. Psychiatry* 59 546–554 10.1016/j.biopsych.2005.07.031 16256957

[B121] Pagsberg A. K. Baare W. F. C. Christensen A. M. R. Fagerlund B. Hansen M. B. LaBianca J. (2007). Structural brain abnormalities in early onset first-episode psychosis. *J. Neural Transm.* 114 489–498 10.1007/s00702-006-0573-8 17024324

[B122] Palha J. A. Goodman A. B. (2006). Thyroid hormones and retinoids: a possible link between genes and environment in schizophrenia. *Brain Res. Rev.* 51 61–71 10.1016/j.brainresrev.2005.10.001 16325258

[B123] Pantelis C. Velakoulis D. McGorry P. D. Wood S. J. Suckling J. Phillips L. J. (2003). Neuroanatomical abnormalities before and after onset of psychosis: a cross-sectional and longitudinal MRI comparison. *Lancet* 361 281–288 10.1016/S0140-6736(03)12323-9 12559861

[B124] Pardey M. C. Kumar N. N. Goodchild A. K. Cornish J. L. (2012). Catecholamine receptors differentially mediate impulsive choice in the medial prefrontal and orbitofrontal cortex. *J. Psychopharmacol*. 27 203–212 10.1177/0269881112465497 23135240

[B125] Perlmann T. Jansson L. (1995). A novel pathway for vitamin-a signaling mediated by Rxr heterodimerization with Ngfi-B and Nurr1. *Gene Dev.* 9 769–782 10.1101/gad.9.7.769 7705655

[B126] Piontkewitz Y. Arad M. Weiner I. (2012). Tracing the development of psychosis and its prevention: what can be learned from animal models. *Neuropharmacology* 62 1273–1289 10.1016/j.neuropharm.2011.04.019 21703648

[B127] Prufer K. Barsony J. (2002). Retinoid X receptor dominates the nuclear import and export of the unliganded vitamin D receptor. *Mol. Endocrinol.* 16 1738–1751 10.1210/me.2001-0345 12145331

[B128] Prufer K. Veenstra T. D. Jirikowski G. F. Kumar R. (1999). Distribution of 1,25-dihydroxyvitamin D3 receptor immunoreactivity in the rat brain and spinal cord. *J. Chem. Neuroanat.* 16 135–145 10.1016/S0891-0618(99)00002-2 10223312

[B129] Rakic P. Bourgeois J.-P. Goldman-Rakic P. (1994) “Synaptic development of the cerebral cortex: implications for learning, memory, and mental illness,” in *Progress in Brain Research, The Self-Organizing Brain: From Growth Cones to Functional Networks* eds van Pelt J. Corner M. Uylings H. Lopes F. da Silva (Amsterdam: Elsevier) 227–243 10.1016/S0079-6123(08)60543-9 7800815

[B130] Rutz S. Majchrzak M. Siedschlag V. Barbelivien A. Harati H. Rothmaier A. K. (2009). The modulation of striatal dopamine release correlates with water-maze performance in aged rats. *Neurobiol. Aging* 30 957–972 10.1016/j.neurobiolaging.2007.09.011 17997198

[B131] Sakurada K. Ohshima-Sakurada M. Palmer T. D. Gage F. H. (1999). Nurr1, an orphan nuclear receptor, is a transcriptional activator of endogenous tyrosine hydroxylase in neural progenitor cells derived from the adult brain. *Development* 126 4017–4026 10457011 10.1242/dev.126.18.4017

[B132] Seeman P. Kapur S. (2000). Schizophrenia: more dopamine, more D2 receptors. *Proc. Natl. Acad. Sci. U.S.A.* 97 7673–7675 10.1073/pnas.97.14.7673 10884398 PMC33999

[B133] Seeman P. Lee T. (1975). Antipsychotic drugs: direct correlation between clinical potency and presynaptic action on dopamine neurons. *Science* 188 1217–1219 10.1126/science.1145194 1145194

[B134] Shotbolt P. Stokes P. R. Owens S. F. Toulopoulou T. Picchioni M. M. Bose S. K. (2011). Striatal dopamine synthesis capacity in twins discordant for schizophrenia. *Psychol. Med.* 41 2331–2338 10.1017/S0033291711000341 21426628

[B135] Smidt M. P. Burbach J. P. (2007). How to make a mesodiencephalic dopaminergic neuron. *Nat. Rev. Neurosci.* 8 21–32 10.1038/nrn2039 17180160

[B136] Smith Y. Kieval J. Z. (2000). Anatomy of the dopamine system in the basal ganglia. *Trends Neurosci.* 23 S28–S33 10.1016/S1471-1931(00)00023-9 11052217

[B137] Spear L. P. (2000). The adolescent brain and age-related behavioral manifestations. *Neurosci. Biobehav. Rev.* 24 417–463 10.1016/S0149-7634(00)00014-2 10817843

[B138] Sulzer D. Maidment N. T. Rayport S. (1993). Amphetamine and other weak bases act to promote reverse transport of dopamine in ventral midbrain neurons. *J. Neurochem.* 60 527–535 10.1111/j.1471-4159.1993.tb03181.x 8419534

[B139] Sutherland M. K. Somerville M. J. Yoong L. K. Bergeron C. Haussler M. R. McLachlan D. R. (1992). Reduction of vitamin D hormone receptor mRNA levels in Alzheimer as compared to Huntington hippocampus: correlation with calbindin-28k mRNA levels. *Brain Res. Mol. Brain Res.* 13 239–250 10.1016/0169-328X(92)90032-7 1317496

[B140] Swerdlow N. R. Stephany N. Wasserman L. C. Talledo J. Sharp R. Auerbach P. P. (2003). Dopamine agonists disrupt visual latent inhibition in normal males using a within-subject paradigm. *Psychopharmacology* 169 314–320 10.1007/s00213-002-1325-6 12610717

[B141] Tagami T. Lutz W. H. Kumar R. Jameson J. L. (1998). The interaction of the vitamin D receptor with nuclear receptor corepressors and coactivators. *Biochem. Biophys. Res. Commun.* 253 358–363 10.1006/bbrc.1998.9799 9878542

[B142] Torrey E. F. Bowler A. E. Clark K. (1997a). Urban birth and residence as risk factors for psychoses: an analysis of 1880 data. *Schizophr. Res.* 25 169–176 10.1016/S0920-9964(97)00020-0 9264172

[B143] Torrey E. F. Miller J. Rawlings R. Yolken R. H. (1997b). Seasonality of births in schizophrenia and bipolar disorder: a review of the literature. *Schizophr. Res.* 28 1–38 10.1016/S0920-9964(97)00092-3 9428062

[B144] Turner K. M. Young J. W. McGrath J. J. Eyles D. W Burne T. H. J. (2013). Cognitive performance and response inhibition in developmentally vitamin D (DVD)-deficient rats. *Behav. Brain Res.* 242 47–53 10.1016/j.bbr.2012.12.029 23275047

[B145] Veenstra T. D. Prufer K. Koenigsberger C. Brimijoin S. W. Grande J. P. Kumar R. (1998). 1,25-Dihydroxyvitamin D-3 receptors in the central nervous system of the rat embryo. *Brain Res.* 804 193–205 10.1016/S0006-8993(98)00565-4 9757035

[B146] Veling W. Hoek H. W. Selten J. P. Susser E. (2011). Age at migration and future risk of psychotic disorders among immigrants in the Netherlands: a 7-Year incidence study. *Am. J. Psychiatry* 168 1278–1285 10.1176/appi.ajp.2011.11010110 22193672

[B147] Volpicelli F. Perrone-Capano C. Da Pozzo P. Colucci-D’Amato L di Porzio U. (2004). Modulation of nurr1 gene expression in mesencephalic dopaminergic neurones. *J. Neurochem.* 88 1283–1294 10.1046/j.1471-4159.2003.02254.x 15009684

[B148] Voorn P. Kalsbeek A. Jorritsmabyham B. Groenewegen H. J. (1988). The prenatal and postnatal-development of the dopaminergic cell groups in the ventral mesencephalon and the dopaminergic innervation of the striatum of the rat. *Neuroscience* 25 857–887 10.1016/0306-4522(88)90041-3 3405431

[B149] Vuillermot S. Weber L. Feldon J. Meyer U. (2010). A longitudinal examination of the neurodevelopmental impact of prenatal immune activation in mice reveals primary defects in dopaminergic development relevant to schizophrenia. *J. Neurosci.* 30 1270–1287 10.1523/JNEUROSCI.5408-09.2010 20107055 PMC6633802

[B150] Wallen-Mackenzie A. de Urquiza A. M. Petersson S. Rodriguez F. J. Friling S. Wagner J. (2003). Nurr1-RXR heterodimers mediate RXR ligand-induced signaling in neuronal cells. *Gene Dev.* 17 3036–3047 10.1101/gad.276003 14681209 PMC305256

[B151] Wallen A. Zetterstrom R. H. Solomin L. Arvidsson M. Olson L. Perlmann T. (1999). Fate of mesencephalic AHD2-expressing dopamine progenitor cells in Nurr1 mutant mice. *Exp. Cell Res.* 253 737–746 10.1006/excr.1999.4691 10585298

[B152] Wallen A. Castro D. S. Zetterstrom R. H. Karlen M. Olson L. Ericson J. (2001). Orphan nuclear receptor Nurr1 is essential for Ret expression in midbrain dopamine neurons and in the brain stem. *Mol. Cell. Neurosci.* 18 649–663 10.1006/mcne.2001.1057 11749040

[B153] Wang Y. Chiang Y. H. Su T. P. Hayashi T. Morales M. Hoffer B. J. (2000). Vitamin D-3 attenuates cortical infarction induced by middle cerebral arterial ligation in rats. *Neuropharmacology* 39 873–880 10.1016/S0028-3908(99)00255-5 10699453

[B154] Weinberger D. R. (1987). Implications of normal brain-development for the pathogenesis of schizophrenia. *Arch. Gen. Psychiatry* 44 660–669 10.1001/archpsyc.1987.01800190080012 3606332

[B155] Westerink B. H. C. (1985). Sequence and significance of dopamine metabolism in the rat-brain. *Neurochem. Int.* 7 221–227 10.1016/0197-0186(85)90108-1 20492917

[B156] Wieczorek W. J. Kruk Z. L. (1994). Differential action of (+)-amphetamine on electrically-evoked dopamine overflow in rat-brain slices containing corpus striatum and nucleus-accumbens. *Brit. J. Pharmacol.* 111 829–836 10.1111/j.1476-5381.1994.tb14813.x 8019759 PMC1910092

[B157] Willins D. L. Narayanan S. Wallace L. J. Uretsky N. J. (1993). The Role of dopamine and ampa kainate receptors in the nucleus-accumbens in the hypermotility response to Mk801. *Pharmacol. Biochem. Behav.* 46 881–887 10.1016/0091-3057(93)90217-H 8309969

[B158] Winter C. Djodari-Irani A. Sohr R. Morgenstern R. Feldon J. Juckel G. (2009). Prenatal immune activation leads to multiple changes in basal neurotransmitter levels in the adult brain: implications for brain disorders of neurodevelopmental origin such as schizophrenia. *Int. J. Neuropsychopharmacol.* 12 513–524 10.1017/S1461145708009206 18752727

[B159] Yoon E. H. Lee K. J. Kim Y. S. Chang M. S. Lee S. H. Park C. H. (2010). Retinoid X receptor a acts as alpha negative regulator in Nurr1-induced dopaminergic differentiation in rat neural precursor cells. *Neuroreport* 21 1162–1166 10.1097/WNR.0b013e328340ccf9 22066143

[B160] Zehnder D. Bland R. Williams M. C. McNinch R. W. Howie A. J. Stewart P. M. (2001). Extrarenal expression of 25-hydroxyvitamin D-3-1 alpha-hydroxylase. *J. Clin. Endocrinol. Metab.* 86 888–894 10.1210/jc.86.2.888 11158062

[B161] Zetterstrom R. H. Solomin L. Jansson L. Hoffer B. J. Olson L. Perlmann T. (1997). Dopamine neuron agenesis in Nurr1-deficient mice. *Science* 276 248–250 10.1126/science.276.5310.248 9092472

